# Evaluation of molecular typing of foodborne pathogens in European reference laboratories from 2012 to 2013

**DOI:** 10.2807/1560-7917.ES.2016.21.50.30429

**Published:** 2016-12-15

**Authors:** Susanne Schjørring, Taina Niskanen, Mia Torpdahl, Jonas T Björkman, Eva Møller Nielsen

**Affiliations:** 1 Unit of foodborne infections, Department of Microbiology and Infection Control, Statens Serum Institut (SSI), Copenhagen, Denmark; 2 European Programme for Public Health Microbiology Training (EUPHEM), European Centre for Disease Prevention and Control (ECDC), Stockholm, Sweden; 3 Food and Waterborne Diseases and Zoonoses Programme, European Centre for Disease Prevention and Control (ECDC), Stockholm, Sweden

**Keywords:** surveillance, PFGE, MLVA, *Salmonella*, VTEC, *Listeria*

## Abstract

In 2012, the European Centre for Disease Prevention and Control (ECDC) initiated external quality assessment (EQA) schemes for molecular typing including the National Public Health Reference Laboratories in Europe. The overall aim for these EQA schemes was to enhance the European surveillance of food-borne pathogens by evaluating and improving the quality and comparability of molecular typing. The EQAs were organised by Statens Serum Institut (SSI) and included *Salmonella enterica* subsp. enterica, verocytotoxin-producing *Escherichia coli* (VTEC) and *Listeria monocytogenes*. Inter-laboratory comparable pulsed-field gel electrophoresis (PFGE) images were obtained from 10 of 17 of the participating laboratories for *Listeria*, 15 of 25 for *Salmonella*, but only nine of 20 for VTEC. Most problems were related to PFGE running conditions and/or incorrect use of image acquisition. Analysis of the gels was done in good accordance with the provided guidelines. Furthermore, we assessed the multilocus variable-number tandem repeat analysis (MLVA) scheme for *S.* Typhimurium. Of 15 laboratories, nine submitted correct results for all analysed strains, and four had difficulties with one strain only. In conclusion, both PFGE and MLVA are prone to variation in quality, and there is therefore a continuous need for standardisation and validation of laboratory performance for molecular typing methods of food-borne pathogens in the human public health sector.

## Introduction

Salmonellosis, verocytotoxin-producing *Escherichia coli* (VTEC) infections and listeriosis are some of the most commonly reported zoonotic diseases within the European Union (EU) [1]. Since 2006, the European Centre for Disease Prevention and Control’s (ECDC) Food- and Waterborne Diseases and Zoonoses (FWD) Programme has been responsible for the EU-wide surveillance of salmonellosis, VTEC infections and listeriosis including the facilitation of the detection and investigation of food-borne outbreaks. Phenotypic parameters of the isolated pathogens are reported by the EU Member States to The European Surveillance System (TESSy) and molecular typing data are reported to the molecular surveillance service within TESSy [2]. 

In view of the surveillance objectives, ECDC has developed a set of specific principles and prerequisites for the systematic incorporation of molecular typing data into routine EU-level surveillance [3,4]. One of the principles includes that the use of internationally agreed molecular typing methods is supported by external quality assessment (EQA) schemes to enhance data quality and comparability. For three food-borne pathogens, namely *Salmonella*, VTEC and *Listeria,* globally agreed standard molecular typing methods, namely pulsed-field gel electrophoresis (PFGE) and multilocus variable-number tandem repeat analysis (MLVA) [5] enable a comparison with isolates from food/feed and animals. 

PFGE is used widely for surveillance [6-8] and outbreak investigations of all three pathogens [9-11]. It is the only generic method for typing of all *Salmonella* serovars and *Listeria* serotypes and global protocols have been developed and standardised by the United States (US) Centers for Disease Control and Prevention (CDC) [12,13]. 

MLVA is serotype specific, and has been developed for *S.* Typhimurium [14,15] with standardisation by the use of reference strains [16]. The method has a higher discrimination power compared with PFGE for *S.* Typhimurium and is widely used for surveillance [17,18] and outbreak investigations [19,20]. 

PFGE and MLVA methods have been standardised in order to allow comparable results across laboratories [12,13,21,22], thus the FWD network decided to use those for developing molecular surveillance at EU level.

This study presents the results from the first round (2012–2013) of the EQAs for molecular typing of *Salmonella,* VTEC and *Listeria monocytogenes* in National Public Health Reference Laboratories (NPHR-Ls) in EU/European Economic Area (EEA) countries and EU candidate countries. The objectives of the EQAs were to assess the quality and comparability of PFGE and MLVA results from participating laboratories.

## Methods

### Organisation

The EQAs were funded by ECDC and organised by Statens Serum Institut (SSI), Denmark. One NPHR-L from each of the 31 EU/EEA countries and four EU candidate countries in 2012–2013 were invited to participate in each of the three EQA schemes (one scheme for each bacterial species). Some countries have different NPHR-L for each species and some countries have one NPHR-L responsible for all three species. The EQA schemes and their different components were optional and laboratories could chose to only participate in selected parts (e.g. only submitting a PFGE gel without performing the analysis of the gel). The *Salmonella* EQA included PFGE, MLVA and phage typing, the VTEC EQA included PFGE, serotyping, genotyping (including subtyping and virulence genes) and phenotypic tests and the *Listeria* EQA included PFGE and serotyping. All details of the EQAs are published as technical reports by ECDC [23-25]. Only the molecular typing results are presented here.

### Strains

For the PFGE parts of the EQAs, bacterial strains (10 *Salmonella,* 10 *Listeria* and 11 VTEC) were selected based on their relevance for the epidemiological situation in Europe, including in recent outbreaks. The serotypes included for PFGE were for *Salmonella*: Aberdeen, Dublin, Enteritidis, Infantis, Mbandaka, Poona, Saintpaul, Strathcona, and Typhimurium (2 strains), for VTEC: O26:H11, O41:H26, O103:H2, O104:H4, O111:H8/H-, O121:H9, O146:H21, O177:H25, O157:H7 (2 strains), O166:H15, for *Listeria*: 1/2a (2 strains), 1/2b (1 strain), 1/2c (3 strains), 4a/4c (1 strain) and 4b (3 strains). For the MLVA part of the *Salmonella* EQA, a total of 10 different representative *S*. Typhimurium strains were selected. 

All the strains included in either MLVA or PFGE were stability tested, blinded and packed for distribution according to the International Standard ISO/IEC 17043:2010 (appendix B.5) [26]. In addition to these strains, reference strains for the different assays were delivered to participants upon request. These included the PFGE reference strain *S.* Brandrup and/or 33 MLVA reference strains, consisting of an original set of 31 previously described MLVA reference strains [16] as well as the recently added STm-SSI32: (3,17,21,18,311) and STm-SSI33: (2,13,9,11,112) strains. The participants were also provided a detailed study protocol specifying all suggested standardised methods for each of the specific species. Moreover, a pre-configured BioNumerics (BN) database with experiment settings and a guide for creating a new database was also made available to them if their BN software was older than version 5. Furthermore, guidance on how to export the BN analysis of PFGE data was provided as well as an Excel sheet converting the obtained MLVA fragment sizes to true allele numbers based on the results obtained when analysing the 33 MLVA reference strains.

### Testing

The participants were instructed to use the Standard PulseNet PFGE protocol for *Salmonella*, VTEC O157 [27] and *Listeria monocytogenes* [28]. For the *S.* Typhimurium MLVA, the *S.* Typhimurium MLVA Standard protocol was suggested [29].

### Data analysis

For PFGE, the data were evaluated as two separate parts (i) the quality of gels and (ii) the quality of the further gel analysis. (i) The gel quality was evaluated according to the ECDC FWD MolSurv Pilot - SOPs 1.0 - Annex 5 - PulseNet US protocol PFGE Image Quality Assessment (TIFF Quality Grading Guidelines) [23-25], by scoring the gel with respect to seven parameters (image acquisition and running conditions, cell suspension, bands, lanes, restriction, gel background, and DNA degradation). (ii) The participant’s ability to perform gel analysis was evaluated separately from the evaluation of the gel quality. However, the gel analysis (part ii) was based on the gels produced in the respective laboratories and therefore the outcome of a participating laboratory’s band assignment is to some degree influenced by its gel quality (part i). The gel analysis (ii) was evaluated by scoring five parameters (position of gel, strips, curves, normalisation, and band assignment) using the BN gel analysis quality guidelines, developed by SSI. All parameters were scored between 1 and 4: 1 (poor), 2 (fair), 3 (good), and 4 (excellent). The evaluation of the participating laboratories’ gel analysis was independently carried out by two experts in PFGE, who subsequently discussed and agreed upon the scores.

The MLVA typing results were scored as correct or incorrect for each strain and the percentage of correct answers was used as the score for each participant.

## Results

### Participation

In total, 35 countries were invited to participate in each EQA. The highest number of laboratories participating was in the PFGE EQA for *Salmonella* and VTEC with 25 and 20 NPHR-L, respectively, compared with 17 for *Listeria*. The number of participants that submitted a PFGE gel (without analysis) were 11/25 for *Salmonella*, 8/20 for VTEC and 4/17 for *Listeria*. The number of laboratories analysing their PFGE gels and submitting export files according to the instructions were 14/25 for *Salmonella,* 12/20 for VTEC and 13/17 for *Listeria* (Table 1). Fifteen laboratories participated in the MLVA part of the *Salmonella* EQA.

**Table 1:** Number of national public health reference laboratories (NPHR-L) submitting external quality assessment (EQA) results by pathogen and method, European Union/European Economic Area, 2012–2013

Pathogen	Number of NPHR-L participating to the MLVA EQA	Number of NPHR-L participating to the PFGE EQA			TOTAL
		PFGE gel only^a^	PFGE gel + analysis^b^	Total	
*Salmonella*	15	11	14	25	27^c^
VTEC	NA	8	12	20	20
*Listeria*	NA	4	13	17	17

MLVA: multilocus variable-number tandem repeat analysis; NA: not applicable; NPHR-L: national public health reference laboratories; PFGE: pulsed-field gel electrophoresis; VTEC: verocytotoxin-producing *Escherichia coli.*

^a^ Submitting a TIFF file of the PFGE profile.

^b^ Analysing the gel profile and submitting export files.

^c^ Two NPHR-L did not participate in the PFGE part of the external quality assessment, but only in MLVA.

### Pulsed-field gel electrophoresis (PFGE)

#### 

##### Gel quality

The majority (61/62) of the submitted results were profiles recognisable as the profile for the relevant EQA strain, i.e. indicating that the laboratory had not by mistake interchanged strains. One laboratory seemed to have exchanged one PFGE VTEC strain with one VTEC strain for the phenotypic tests.

The average scores of all laboratories by parameter and pathogen are listed in Table 2, along with the conditions for being graded an excellent score. For all three pathogens and for four of the seven parameters, the gel quality was good, scoring on average 3.0 or above (Table 2). For VTEC gels, the parameter ‘gel background’ was only 2.9 on average as 3/20 gels were scored ‘poor’, mostly due to large amount of debris in the gels – which can be easily prevented. None of the *Salmonella* or *Listeria* gels obtained the lowest score for this parameter.

**Table 2:** Average pulsed-field gel electrophoresis (PFGE) gel quality scores of laboratories participating in a typing external quality assessment (EQA), by parameters and pathogen, European Union/European Economic Area, 2012–2013

Parameters	Conditions for excellent score	*Salmonella* (n = 25)	VTEC (n = 20)	*Listeria* (n = 17)
Image acquisition and running conditions	Wells included, bottom band 1.5 cm from edge Spacing of standard match global standards	2.6	2.2	2.1
Cell suspension	Even distribution of DNA	3.9	3.5	3.8
Bands	Clear and distinct bands	2.9	2.2	2.5
Lanes	Straight lanes	3.7	3.6	3.8
Restriction	Complete restriction in all lanes	3.6	3.2	3.5
Gel background	Clear background	3.3	2.9	3.2
DNA degradation	No degradation	3.3	3.1	3.2

VTEC: verocytotoxin-producing *Escherichia coli*.

The scores 1 (poor), 2 (fair), 3 (good), and 4 (excellent) were given according to the TIFF Quality Grading Guidelines [23-25].

**Figure 1:**
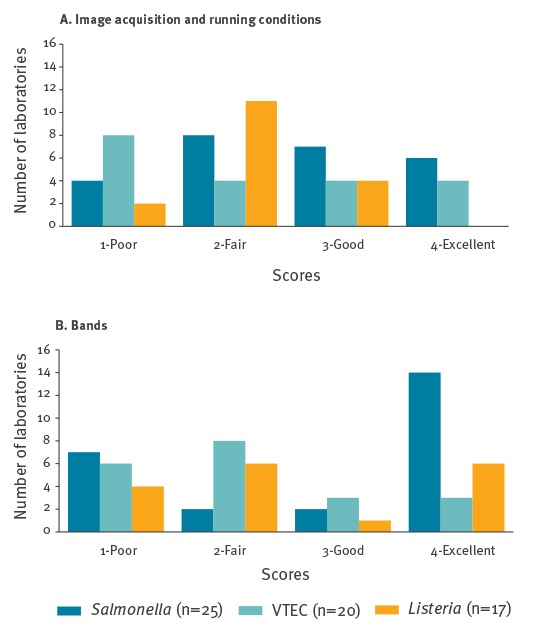
Number of laboratories according to their pulsed-field gel electrophoresis (PFGE) gel quality scores for the parameters a) ‘image acquisition and running conditions’ and b) ‘bands’, European Union/European Economic Area, 2012–2013

**Figure 2:**
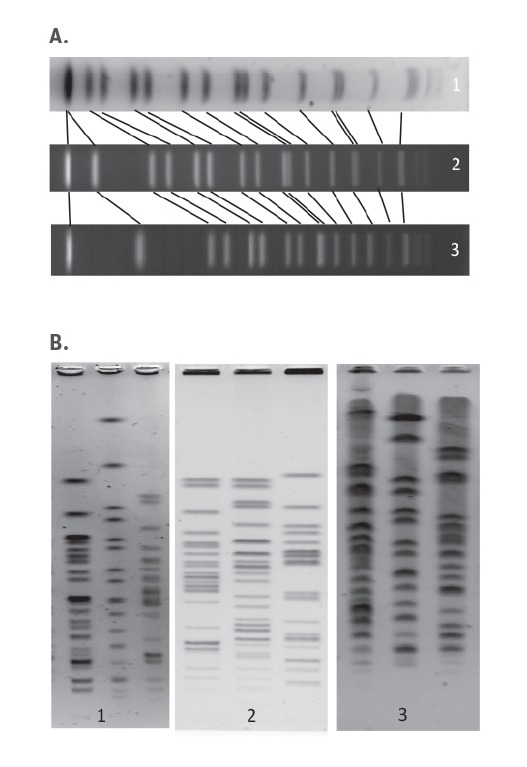
Examples of gel selections with a) incorrect running conditions and b) fuzzy/thick bands

For all pathogens, with respect to the two important parameters ‘image acquisition and running conditions’ and ‘bands’ the average gel quality was only fair (between 2.1 and 2.9). Very diverse individual scores were obtained for these parameters (Figure 1a and 1b). Critical scores (1 or 2 ~ poor or fair) for the parameter ‘image acquisition and running conditions’ were given to 12/25, 12/20 and 13/17 of the gels of *Salmonella*, VTEC and *Listeria* (Figure 1a), respectively. Correct running conditions and thereby the correct spacing of the global standard is crucial for the possibility of inter-laboratory comparison. Incorrect spacing of the standard was more frequently observed on the VTEC gels than the *Salmonella* and *Listeria* gels. For the parameter ‘bands’, clear and distinct bands were seen on 14/25 of the *Salmonella* gels, 6/17 of the *Listeria* gels and only 3/20 of the VTEC gels scored ‘excellent’ (Figure 1b). In addition, 7/25 of the *Salmonella*, 6/20 of the VTEC and 4/17 of the *Listeria* gels obtained the score ‘poor’ (Figure 1b), which indicates that further analysis of the gel was impossible and generally it would be difficult or impossible to compare with profiles on other gels. Examples of submitted gels of poor quality are shown in Figure 2.

VTEC: verocytotoxin-producing *Escherichia coli*.

Each parameter is evaluated and presented separately.

The middle examples (2) scored ‘excellent’ (i.e. score = 4) in all parameters.

Since a low quality score in just one parameter has a high impact on the ability to further analyse the image, the overall across-parameter results showed that inter-laboratory comparable PFGE images could only be obtained from 10 of 17 of the participating laboratories for *Listeria*, 15 of 25 for *Salmonella*, but only nine of 20 for VTEC.

##### Gel analysis

In the PFGE part of the EQAs, involving *Listeria*, *Salmonella* and VTEC, 17 to 25 laboratories per pathogen participated (Table 1) by submitting raw gel images (TIFF files). Depending on the pathogen, between 12 and 15 laboratories also analysed their gels and submitted the results in the form of export files (Table 1). However, one laboratory’s submission was excluded in the *Salmonella* EQA due to incompatibility between the BN versions 6.0 and 7.0, i.e. 14 datasets were included in the gel analysis. Gel analysis was graded on five parameters. The average gel analysis quality scores of each parameter and EQA are listed in Table 3.

**Table 3:** Average gel analysis quality scores of laboratories participating in a typing external quality assessment (EQA), by parameter and pathogen, European Union/European Economic Area, 2012–2013

Parameters	Conditions for excellent score	*Salmonella* (n = 14)^a^	VTEC (n = 12)	*Listeria* (n = 13)
Position of gel	Placement of gel in the frame, inverted	3.5	3.5	3.1
Strips	All lanes correctly defined	4.0	3.8	3.5
Curves	1/3 of the lanes is used for averaging of curve thickness	3.6	3.4	3.5
Normalisation	All bands (incl. below 33kb) assigned correctly in all reference lanes	3.4	2.8	3.2
Band assignment	Bands assigned correctly according to gel quality	3.3	3.3	3.3

The average score of the participating laboratories is presented for each pathogen, and for each of the five parameters. The scores 1 (poor), 2 (fair), 3 (good), and 4 (excellent) were given according to the BioNumerics gel analysis Quality Guidelines [23-25].

^a^ For *Salmonella* 15 laboratories analysed their gels, however one laboratory’s submission was excluded due to incompatibility between the BN versions 6.0 and 7.0.

Laboratories received high scores for all three pathogens on the parameters ‘strips’ and ‘curves’ (Table 3). For both *Salmonella* and VTEC, high scores were also obtained on the parameter ‘position of gel’ but the score was a bit lower for *Listeria*. Two laboratories failed to place the frame below the wells and this had critical influence when the gel was normalised. With regard to the parameter ‘normalisation’, the participants in the VTEC EQA were graded lower than *Salmonella* and *Listeria* with an average of 2.8 because of incorrect band assignment of the reference lanes or failure to include the reference strains in the export files. The average scores of the parameter ‘band assignment’ were equal for all three pathogens (Table 3).

### Multilocus variable-number tandem repeat analysis (MLVA) of *Salmonella* Typhimurium

Of the 15 laboratories that participated in the MLVA part of the EQA, nine laboratories were able to correctly MLVA type all ten EQA strains. Four laboratories reported the correct MLVA profile for nine of the strains, one laboratory had correct results for seven strains, and one for five strains. The typical error accounting for the vast majority of incorrect profiles by these six laboratories was to either replace an absent (NA) locus with a repeat number or vice versa. One of the laboratories seemed to have analysed/reported the MLVA profile for one EQA strain under two strain numbers, thereby obtaining an incorrect profile for one strain. One laboratory had multiple allele errors in several MLVA profiles and these were probably caused by incorrect or lack of calibration of the measured fragment sizes. Table 4 shows the number of laboratories able to submit the correct MLVA profile per strain.

**Table 4:** MLVA profiles of 10 *Salmonella* Typhimurium strains used in a typing external quality assessment (EQA) and number of laboratories assigning an incorrect, accepted and correct MLVA profile to each strain, European Union/European Economic Area, 2012–2013 (n = 15 participating laboratories)

Strain number	MLVA profile					Result categories of the MLVA analysis with number of laboratories per result category			
	STTR9	STTR5	STTR6	STTR10	STTR3	Incorrect^a^	Accepted^b^	Correct	Total correct
**11**	2	9	15	5	212	2	0	13	**13**
**12**	3	12	9	NA	211	0	1	14	**15**
**13**	3	13	NA	NA	211	2	1	12	**13**
**14**	4	14	12	8	211	0	0	15	**15**
**15**	3	16	15	23	311	2	1	12	**13**
**16**	4	18	NA	10	212	1	2	12	**14**
**17**	3	16	NA	NA	311	1	0	14	**14**
**18**	3	13	16	14	311	1	0	14	**14**
**19**	4	8	19 (18)	10	211	2	10	6	**16^c^**
**20**	2	17	NA	15	212	1	0	14	**14**

MLVA: multiple-locus variable-number tandem repeat analysis; NA: not applicable (locus not present) [16].

^a^ Incorrect profiles have repeat change in one or more loci, the exception being ‘accepted profiles’.

^b^ Accepted profiles have one repeat change in one of the highly variable loci STTR5, STTR6 or STTR10.

^c^ The total number of laboratories for this strain is higher than the number of participating laboratories (n=15) because three laboratories correctly identified two MLVA profiles for strain 19, STTR6: 18 (accepted) /19 (correct).

In less stable loci: STTR5, STTR6 or STTR10 [18], the reporting of one repeat change was evaluated as an acceptable result. For one of the EQA-strains, strain ID 19, the STTR6 locus seemed to have changed immediately before shipment resulting in the presence of two alleles in some of the culture vials. This is clear from the variability in results obtained for this locus of strain 19 (data not shown). Both alleles were evaluated as correct.

## Discussion

The EQA schemes for typing of *Salmonella,* VTEC and *Listeria* organised for the NPHR-Ls in the EU/EEA were the first ones specifically including globally agreed molecular typing methods.

Evaluation of the PFGE gel quality showed that the laboratories generally obtained acceptable scores (‘fair’ or above) for the parameters ‘cell suspension’, ‘lanes’, ‘restriction’, ‘gel background’, and ‘DNA degradation’. These parameters were therefore not the most problematic, but it is still desirable to improve the laboratories’ capacity in these areas. However, many laboratories had problems with the critical parameter ‘image acquisition and running conditions’ as well as the parameter ‘bands’. Incorrect running conditions will make it impossible to compare the PFGE profiles with profiles from others gels. It is important to ensure that the running conditions (switch time, buffer temperature, gel material etc.) are as described for the relevant organism, as these vary significantly between species. Generally, the *Salmonella* and *Listeria* gels had a higher quality than the VTEC gels. This is probably due to the fact that PFGE is a less used method in laboratories specialised in VTEC.

Many laboratories seemed to increase the contrast at image acquisition in order to enhance weak bands. Unfortunately, that resulted in thicker bands and made it hard to distinguish double bands. This, together with overloading plugs with DNA, mostly contributed to the low scores for the parameter ‘bands’. In general, it is highly recommendable to take the time to get familiar with the image acquisition equipment and ensure its maintenance as well as the maintenance of the electrophoresis equipment. Several laboratories probably produced a high quality gel, but failed to document this due to poor image capturing.

The grading guidelines indicate that the score ‘fair’ can be obtained for the parameter ‘image acquisition and running conditions’ even when the band spacing of the standard does not match the global standard. In such cases, the score depends on other criteria included in the evaluation of this parameter. This is, however, inappropriate as it gives the impression that a gel that cannot be normalised correctly is still acceptable. In this EQA, some of the gels that obtained the score ‘fair’ for all parameters were not suitable for inter-laboratory comparison. Therefore, in the coming EQAs the scoring system will be modified to ensure that a gel with such severe quality deficiencies, that it is impossible to reliably compare with gels obtained in other laboratories, is scored ´poor´ in the relevant parameters. In general, an acceptable quality should be obtained for each parameter since a low quality score in just one parameter can have a high impact on the ability to further analyse the image and compare it with other profiles.

On average, 65% (40/62) of laboratories that performed PFGE on the different pathogens conducted also the subsequent gel analysis, i.e. the normalisation and band assignment that provides the actual PFGE profiles for comparison. This analysis requires the use of a specialised software, BN, which some laboratories might not have access to or limited experience with for PFGE analysis. However, to be able to carry out national surveillance and submit profiles to TESSy, it is important to have the capacity to analyse and interpret the PFGE gels, as submission of raw TIFF images to TESSy is not possible. Correct normalisation of the gel is critical for inter-laboratory comparability. The ability to normalise a gel according to an international standard depends on the use of standard running conditions and reference strains (as evaluated by the scoring parameter ‘image acquisition and running conditions‘ from the TIFF Quality Grading Guidelines) as well as the correct use of the reference lanes for normalisation in the further analysis of the gel using the BN software. Standardisation of band assignment is difficult since the ability to recognise and distinguish bands (e.g. the presence of double bands, weak bands, etc.) is highly dependent on gel quality. In these EQAs, focus was on increasing the laboratories’ ability to produce high quality PFGE gels that can be normalised and compared when submitted to a shared database. The participants’ ability to assign bands on their produced gels was also evaluated; however, the large variability in gel quality across laboratories made it difficult to classify profiles into definitive types. Therefore, we did not evaluate the performance in relation to participants’ ability to assign a standard nomenclature. The main goal was to obtain a sufficient gel quality and normalisation for comparison in a centralised and curated database, where the nomenclature is centrally assigned by the curators of the database. In future EQAs, the ability to perform standardised band assignment could be evaluated by providing images of high quality PFGE gels to the participants. One of the challenges for standardisation within EU is that standard protocols can only be recommended. In order to include as many laboratories as possible, ECDC decided that it was not possible to make protocols mandatory for this EQA. In the public health sector within the EU there are no obligatory methods when uploading to TESSy, in contrast to other networks such as PulseNet in the US where the use of standardised methods are mandatory.

Fifteen laboratories participated in the MLVA part of the EQA, which consisted of typing ten strains of *S*. Typhimurium including monophasic variants of this serovar. Of the 15 laboratories, nine typed all MLVA strains correctly and an additional four reported correct MLVA profiles for nine strains. One laboratory had major problems with the correct allele calling. Except for this one laboratory that seemed to have general problems with the calibration of fragment sizes, most other errors were related to overlooking the presence of a locus (reporting as absent allele where a fragment should have been detected) or reporting an allele number for an absent locus. This can be due to the use of an unbalanced primer mix resulting in variability in peak heights and thereby either missing a peak or misidentifying background noise for a signal. Another explanation can be that the samples for capillary electrophoresis were overloaded, which can cause large peaks to pick up other primer dyes used in the mix and thereby be mistaken for a peak representing another locus.

One of the EQA test strains had a mix of alleles in the cultures sent to at least some of the laboratories. Three laboratories were impressively able to find both alleles and submit the results. For a highly discriminatory method like MLVA, there is always a risk of changes occurring in the strains during the transport and culturing before testing. In general, changes only occur in the fast changing loci STTR5, STTR6 and STTR10 and changes in these loci were therefore accepted when evaluating the results of this EQA. To our knowledge, several of the laboratories participating in the EQA are not performing the MLVA method on a routine basis and we therefore expect that the performance could be even higher with more experience.

This first comprehensive EQA scheme on molecular typing for NPHR-Ls in the EU/EEA provides invaluable information for the development of molecular typing-based surveillance of food-borne infections and gradual implementation of molecular typing in the routine surveillance at the EU level.

The results showed high variation of the typing capabilities between the laboratories, but the results also varied depending on the pathogens and methods. The MLVA results were reassuring with more than half of the laboratories providing correct results for all strains and most of the problems reported were errors in single loci. Mistakes in MLVA profiles submitted to TESSy will have a direct impact on the possibility of detecting clusters as MLVA results are not curated, but used directly for cluster detection and case definition. More MLVA profiles than PFGE profiles of *S*. Typhimurum are submitted to TESSy. The majority of laboratories participating in the *Salmonella* and *Listeria* EQAs were able to produce PFGE profiles that could be compared with profiles from other laboratories. Less than half of the laboratories participating in the VTEC EQA produced images with acceptable quality for comparison and need further improvements before submitting to TESSy. The most common problems were related to the running conditions and use of the image acquisition equipment, which in some cases are easily overcome and related to thick bands. There are no formal requirements regarding the proficiency in PFGE for a NPHR-L to be allowed to submit profiles to TESSy. However, all laboratories submitting to TESSy except one have so far participated in the relevant EQA. The PFGE data in TESSy are curated and poor quality profiles will be marked as ‘rejected’ and not used in the cluster detection unless linked to an ongoing cross-border outbreak. The data submitter will be notified of rejected profiles, but as new improved data are not always submitted, the EU-wide surveillance is influenced by the sub-optimal performance in PFGE. In general, there is a correlation between poor performance in the EQAs and lower quality of gels submitted to TESSy. Therefore, it is important for the European surveillance of food-borne infections that the laboratories use the feedback from the EQA to improve the quality of the molecular typing used for the national surveillance and for submission to TESSy.

The fact that PFGE is laborious, personnel sensitive, and prone to quality variations warrants the need for identifying more robust and reproducible methodologies for the molecular typing-based surveillance in the future. ECDC supports the standardisation of methods that fulfil the criteria for integration into EU level surveillance and follows closely the rapid development of whole genome sequencing techniques in the international scientific community [30]. However, at this point, PFGE and MLVA are still the most widely used methods for food-borne bacterial pathogens in NPHR-Ls in EU. The continued use of PFGE and MLVA in some countries and the parallel introduction of new sequence based methods in other countries pose a challenge for the EU level surveillance. The support of quality improvement in the laboratory procedures and interpretation of results, e.g. sustaining EQA schemes and training courses, will also be important for the inter-laboratory comparability of typing results in the future.
